# What Is Case Management? A Scoping and Mapping Review

**DOI:** 10.5334/ijic.2477

**Published:** 2016-10-19

**Authors:** Sue Lukersmith, Michael Millington, Luis Salvador-Carulla

**Affiliations:** 1 Faculty of Health Sciences, Centre for Disability Research and Policy, University of Sydney, Sydney, Australia; 2 Mental Health Policy Unit, Brain & Mind Centre, University of Sydney, Sydney, Australia

**Keywords:** case management, complexity, care coordination, integrated care, taxonomy, scoping

## Abstract

The description of case management in research and clinical practice is highly variable which impedes quality analysis, policy and planning. Case management makes a unique contribution towards the integration of health care, social services and other sector services and supports for people with complex health conditions. There are multiple components and variations of case management depending on the context and client population. This paper aims to scope and map case management in the literature to identify how case management is described in the literature for key complex health conditions (e.g., brain injury, diabetes, mental health, spinal cord injury). Following literature searches in multiple databases, grey literature and exclusion by health condition, community-based and adequate description, there were 661 potential papers for data extraction. Data from 79 papers (1988–2013) were analysed to the point of saturation (no new information) and mapped to the model, components and activities. The results included 22 definitions, five models, with 69 activities or tasks of case managers mapped to 17 key components (interventions). The results confirm the significant terminological variance in case management which produces role confusion, ambiguity and hinders comparability across different health conditions and contexts. There is an urgent need for an internationally agreed taxonomy for the coordination, navigation and management of care.

## Introduction

Case management, also known as care coordination is a complex integrated health and social care intervention and makes a unique contribution to the health, social care and participation of people with complex health conditions.[1,2,3,4]. In the 1960’s case management emerged in response to the de-institutionalisation of large numbers of people with severe mental health conditions who required referral to outpatient health and other community services. During the 1970’s and 1980’s, the increasing cost of health care and de-centralisation of health services influenced the role of case managers [5,6]. Since the 1990’s, case management has existed in a range of settings including acute, post-acute hospital, rehabilitation, long term care and community-based settings. Case management tasks are now performed by people from various disciplines, for people with different problems in diverse contexts and communities. These multiple interdependent and interacting parameters of case management produce variability in the description of case management [5,7,8,9]. The significant terminological variance, lack of understanding and a common language for case management and care coordination has impeded quality analysis, policy and planning [4,10,11,12]. There is an urgent need for a common international language, but which first requires an understanding of the terms used to describe case management in the literature.

There are multiple parameters that influence case management. Case management operates in very different service sectors (health, social, correctional, work/vocational, veterans, legal sectors) and different settings (public sector, private and non-government organisations) and with different community and support resources (high and low resource settings). Its presence in diverse contexts demonstrates the importance of case management in the horizontal integration of care across health services, social services and other sectors as well as the vertical integration across primary, community, hospital and tertiary health care services [13].

In the health sector, case management and care coordination occurs within an inpatient setting, or mobile and community-based. In this scoping review we only considered community-based case management. Community-based case management is a mobile rather than office based health service. Case manager contact with the client (and/or their family) may occur in a different setting such as the client’s home, workplace or other community venue as considered appropriate by the case manager and client. Community-based case management is the most holistic and person-centred of the approaches (model) as it meets at the junction of the client in their own context. Due to its holistic and comprehensive approach, community-based case management is also likely to involve most of the components of case management of other models that have a narrower focus.

Health sector case managers are from different disciplines (e.g. nursing, occupational therapy, physiotherapy, psychology, rehabilitation counselling, social work, speech pathology) and different practice areas (social and welfare, primary care). Further, there are a number of case management models and theories underpinning practice approaches, due in part to the different sectors where case management operates, the age and health conditions of the client [9]. Hence, both in practice and the literature, a range of names are applied to the role and tasks of a case manager such as: community/care coordinator, support facilitator or broker, case monitor, discharge planner, planning facilitator, case worker, clinical/rehabilitation case manager. Other client characteristics and temporal factors (e.g. whether the client’s problem is new, acute or chronic) also affect the tasks and actions of the case manager. All these different factors related to the case manager, client and context influence what case managers do (i.e. case management tasks as interventions). Whilst there are differences between case management tasks and context, there are also similarities, yet there is no common language to describe these variations.

In spite of the abundance of literature on case management in all its forms, case management descriptors are often non-existent or poorly described with mixed concepts and constructs. There appears to be no consensus on what is, and importantly what is not case management. The heterogeneity, complexity and inadequate descriptions of the components of case management demands a flexible exploratory approach and consideration of a breadth of literature compared to the methods of a focused and narrower systematic review. This review aims to characterise and map how case management has been described in the literature. The review did not seek to assess the quality, nor synthesise the evidence on effectiveness of case management interventions. The focus in this research programme was on the components and definitions. It is the first step of a larger study to develop a taxonomy, a knowledge map and common language for community-based case management. Community-based case management was the focus because it is likely to contain elements of other approaches. People with key complex and chronic health conditions were selected, as case management is frequently used to support their management and the integration of their care.

## Aim of the research

The objective of this study was to scope and map *‘How case management is described in the literature’* in particular the definition, the theoretical basis, the components and activities (interventions) performed by the case manager.

## Theory and Methods

### Study design

The study design was a scoping and mapping review. As exploratory research, scoping reviews are particularly appropriate when the area is complex, and used to map the key concepts underpinning a research area [14]. A scoping study aims *‘to map the literature on a particular topic or research area and provide an opportunity to identify key concepts; gaps in the research; and types and sources of evidence to inform practice, policymaking, and research’* (p. 8) [15]. A scoping review balances the feasibility of the literature search with the breadth and comprehensiveness in the scoping process [16].

The scoping review used five of the six steps in the framework articulated by Arskey et al [17] and extended by Levac [16] which are: 1) identifying the research question; 2) identifying relevant studies; 3) study selection; 4) charting (mapping) the data; 5) collating, summarizing and reporting the results. Consistent with many scoping reviews, quality appraisal was not undertaken as the focus was on language and descriptions of the concepts and components of case management rather than the methodology, outcomes and efficacy of the included studies [15,16,18,19,20].

### Scoping and mapping methodology

We used an iterative process in the scoping review that allowed for flexibility in the search, reviewing and mapping steps. A flexible approach was necessary due to the diversity in the terms around case management, the model or approach taken, the contexts in which it operates and the health conditions of the recipients of case management. The steps taken for the scoping review are outlined below:

#### 1. The research question

The main research question was ‘How was case management described in the literature’. The sub-questions were:

- How was case management for complex and chronic health conditions, described in the literature (brain injury, diabetes, mental health, spinal cord injury)?
- What was the theoretical basis (the model) (if any) linked to the case management approach?
- What were the components, and activities performed in case management; and how are they described?

#### 2. Identify relevant studies

This scoping study used quantitative, qualitative research literature as well as the grey literature. Peer reviewed papers provide information from observational and experimental research. Grey literature provides information from expert practice knowledge and expert experience knowledge [21]. In this study we consider grey literature to be literature ‘*produced at all levels of government, academics, business, industry in print and electronic formats, but which is not controlled by commercial publishers*’ [22]. It includes papers, reports, technical notes or other documents produced and published by governmental agencies, academic institutions, professional associations such as case management societies, and other case management organisations and groups that develop standards or describe services and the activities of case managers.

The search terms and strategy were developed, trialled and discussed then refined with the co-authors and an information specialist. Over three meetings, the co-author team reviewed examples of the literature and refined the selection of studies. This refinement involved combining key words for case management and key words for definition in the final search strategy with limits to specific health conditions. Our decisions on key words and limits are outlined below:

– The variation in names, and complexities of contexts and health conditions posed challenges to systematic searching across multiple databases. We collectively identified the relevant descriptors of case management for the key word search terms based on our familiarity with the literature and community-based case management context.
– There were no limits on the type of study as the range of literature of interest included qualitative, quantitative intervention and non-intervention studies for key health conditions, reports on case management standards, service descriptions, literature reviews and theoretical papers.
– Literature on case management not provided in the community was excluded. However, research papers and grey literature that referred to general case management activities and actions were included.
– The number of descriptions for case management required limits established for the range of health conditions. Five complex or chronic health conditions were included: brain injury, diabetes, mental health conditions and spinal cord injury. Brain injury was included as it is complex health condition and potentially impacts multiple domains of health. It was also of interest to the industry partner (Lifetime Care) involved in the larger study [23]. Mental health conditions were included because of the complex impact of the conditions but also because of its history in case management. Diabetes was included as it is a common chronic health condition. Although less common, spinal cord injury was included as it provides its own set of unique challenges around long term community-based and integrated supports.

Multiple databases were searched for published literature, complemented by searches on key organisation websites and snowballing with hand searching of references lists. The database search was carried out in Week 3 July 2013. The databases were Medline, Cochrane, OTseeker, and PsycBITE. The grey literature key websites searches were conducted in August 2013 and February 2014. The organisational websites were: *Australia*: Case Management Society of Australian and New Zealand (CMSA); Transport Accident Commission (TAC); Lifetime Care and Support Authority (LTC); National Disability Insurance Agency (NDIA); WorkCover Authority (NSW), Brain Injury Rehabilitation Directorate (New South Wales – NSW); Department of Health NSW; *Canada-* National Case Management Network; *United Kingdom* (UK) – Case Management Society of the United Kingdom (CMSUK); British Association of Brain Injury Case Managers (BABICM); National Health Service (NHS); *United States of America* (USA) Agency for Healthcare Research and Quality; Commission for Case Manager Certification; Case Management Health System; Case Management Society of America; American Case Management Association.

The limits were English language, humans with no limits on study type. The inclusion criteria were:

– No limits on publication dates (Medline 1946- Week 2 July 2013)
– Community-based case management
– Case management related to health conditions of brain injury, diabetes, mental health conditions, spinal cord injury
– A definition of the case management and description of the actions, activities, interventions.

#### 3. Study selection

The authors agreed that an iterative process to the exclusion, selection of studies and data extractions was appropriate. In order to manage the copious amounts of literature located, a hierarchy of steps for the exclusion of literature was developed in consultation with co-authors. A bibliographic manager database (EndNote X7) supported the management of the body of literature and exclusion process. The steps for exclusion after the removal of duplicated papers were:

- Exclusion by health conditions, social issues (e.g. ex-prisoners or offenders, homeless persons), single health conditions in low health service resource settings (e.g. Malaria in a developing country),
- Exclusion by case management setting (inpatient, acute care or residential settings such as nursing home, correctional institution), telehealth (no face to face).
- Exclusion because of inadequate (or absence) of a description of case management, the case manager actions or interventions.

#### 4. Mapping the data (charting)

The scoping review involved conceptual mapping to the point of saturation when no new descriptions, concepts or components were identified [17,24]. The focus was on the components and definitions of case management interventions. The information was extracted and stored on an Excel spreadsheet for data management and to enable numerical summation and qualitative analysis. SL extracted data from a sample of 6 papers, which was then reviewed and checked by LSC and MM. The information variables to be extracted were then revised and reduced in agreement with all authors. SL continued with the data extraction and mapping. The final extraction table was reviewed by all authors. Obvious inconsistencies noted were discussed and revisions made.

Extraction and mapping of the case management information began at a global level of the country and type of paper, followed by high level information on the model or approach, theoretical basis, then more detailed components and then finally the description of these components. The final variables mapped were: paper author, year of publication, title, type of study where relevant (or paper), health condition of population, country of study, name of case management model, linked theoretical basis, case management definition, components of case management, descriptors, actions/activities described (sometimes called steps, activities, actions or interventions in the literature) and additional comments. The mapping of information was done to the point of saturation, where no new information (concepts, descriptions, components) were identified. Once it was apparent that no new information was extracted, a further six papers were reviewed and data extracted and mapped, to ensure that the point of saturation had been reached.

#### 5. Collating, summarizing and reporting the results

The information and mapping results from the studies were collated, analysed, summarised and reported. The results were also used as one step in a larger study to develop a taxonomy on case management [23].

## Results

**Figure 1:**
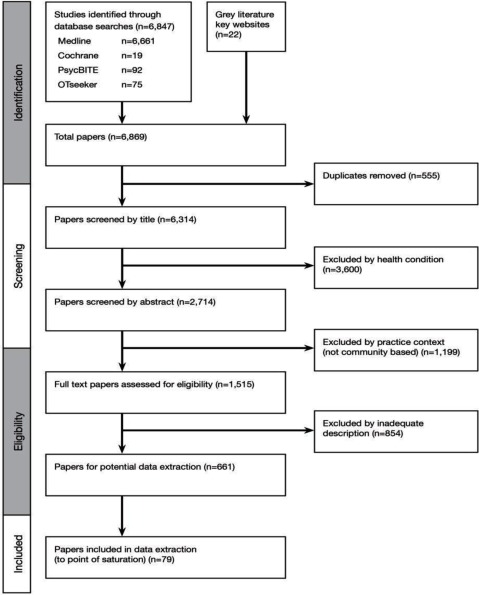
Flow of Study Selection.

Our search yielded a total of 6,847 peer reviewed research study papers and 22 grey literature papers, a total of 6,869 references. This was reduced to 6,314 after duplicates were removed (see Figure 1 for a summary of the screening and eligibility process). After reviewing the titles and abstracts from the search results for health condition (excluded n = 3,600), and removing practice context other than community-based (excluded n = 1,199), and finally removing those with inadequate description in the paper (excluded n = 854), we had 661 potential references for data extraction and mapping. A total of 12 grey literature papers and 61 randomly selected research papers were included in the data extraction and mapping to the point of saturation, when no new information was provided. We selected the grey literature papers because of their focus and the content related to the components to be mapped (model, definition, description of activities or interventions by case managers), such as model descriptions or statements from professional case management associations. To ensure the point of saturation was reached, the data from a further 6 research papers was extracted and mapped making a total of 79 papers.

The papers analysed included 65 papers from peer reviewed journals published 1988–2013 and 14 papers from the grey literature. Appendix 2 provides the details of the 79 included papers. Table 1 describes the global analysis of the papers. In 63 papers there was 10 different countries of focus and 14 there was an international perspective (e.g. literature review). There were 26 papers on mental health, eight on diabetes or chronic/long term health conditions, 12 brain injury, two on spinal cord injury and 31 were not related to specific health conditions. There was one systematic review, 42 qualitative research methods papers, 7 intervention studies, 11 theoretical papers, 5 editorial perspectives or expert opinion, 11 papers were practice guidance and professional association standards and two conference papers.

**Table 1:** Description of the mapped papers.

*Characteristic*	*Source of the paper*	
	Published papers (n = 65)	Grey Literature (n = 14)

**Country of focus**		
*Australia*	7	9
*Canada*	1	1
*Germany*	1	0
*Hong Kong*	1	0
*Japan*	1	0
*New Zealand*	0	1
*Spain*	1	0
*Sweden*	1	0
*United Kingdom*	5	1
*United States America*	33	2
*International*	14	0
**Health Condition**		
*Mental Health*	26	0
*Diabetes/chronic or long term health condition*	8	0
*Brain injury*	8	4
*Spinal Cord Injury*	2	0
*Not specific*	21	10
**Type of paper/study**		
*Systematic review*	1	n/a
*Qualitative study (includes literature review)*	40	2
*Intervention study (includes study protocols)*	7	n/a
*Theoretical paper*	11	n/a
*Editorial/perspective/expert opinion*	5	0
*Practice guidance/standards*	1	10
*Conference paper*	0	2

The next layer of data extraction resulted in an increasing level of detail on case management as described in the literature. Twenty-three specifically identified definitions of case management, (rather than general statements) are provided in Appendix 3. Some definitions were repeated in a number of papers, for example a case management society definition was used in a number of papers.

We found descriptions of different models and theoretical descriptions of the case management approaches in 23 papers. These were mapped to five different models. Exploration on the most common or frequently adopted model was not in the study scope. In some instances, there was a specific model or theoretical basis. For other papers, the approach was broadly described. On this basis, we could map the approach to a model. In other papers several models were discussed (e.g. systematic review). There was a total of 57 papers which did not identify the theoretical basis of the case management approach nor refer to a model. The mapped models of case management, related terms, theoretical description and case management features are provided in Table 2. In this table, we have not provided examples of papers providing a description as many papers such as literature reviews, opinion or theoretical papers and systematic reviews referred to a number of these models or their variations.

**Table 2:** Mapped models of case management, and related names, theoretical description and case management features.

Model and mapped terms	Theoretical description	Case management features

**1. Broker** **– Service broker** **– Managed care** **– Medical case management** **– Generalist** – Gatekeeper [9,25,26,27,28,29]*	An impartial organizational or service focused approach to connect a patient to needed services and to coordinate between different service providers, with an emphasis on a network of providers thereby containing costs by preventing inappropriate access and use of services	Case managers attempt to assist clients to identify their needs and broker services and supports. Contact is limited.
**2. Clinical** **– Rehabilitation** **– Direct care [28,30,31,32,33,34,35,36,37,38,39,40,41,42]**	Involves clinical, collaborative, strategic and communication roles with patient and key stakeholders (e.g. providers, payers, employers): establishes comprehensive case management goals and objectives, interventions, and outcomes including specified timeframes; provides clinical interventions and brokers other clinical services; aims to assist, facilitate, monitor and resolve client issues using clinical skills, clinical services and community resources; may involve adjusting the therapeutic regimen or communicate the need for adjustment to other providers. The individual goals and needs of the client dictate the response and services. In the rehabilitation model this aim is to restore functional ability prior to the injury or illness; case management is extended to include identifying and assessing client skill deficits, barriers to achievement of personal goals, teach skills, provide support and responsibility for the continuity of care and coordinating services including in times of crisis.	Contact can be brief, or an episode of planned activity over 2–3 years.
**3. Chronic care** **– Long term** **– Integrated care** **– Standard [9,43,44,45,46,47]**	More system wide integrated care but tailored to the individual e.g. in primary practice working with a multi-disciplinary team and utilizing system supports. Provides proactive support by the team; and recognizes that quality care is predicated on productive interactions between clients, families and caregivers, providers ; case managers are providers with specific system supports (e.g., protocols), structured relationships with specialist expertise for consultation, support and integration; typically have strong links to the primary care provider to support ongoing coordinated and integrated care with follow-up; condition neutral and is applicable across conditions and risk factors	Longer term involvement with a focus on the integration of care and supports
**4. Strengths based** **[9,27,28,30,38,39,41,48,49,50,51,52,53]**	Based on the premise of the client using their own strengths, resilience, interests, potentials, abilities and knowledge to lead to recovery rather than on their limits (deficits); adopts an ecological perspective that recognizes the importance of people’s environments (context), the individual’s resilience; emphasises the importance of the relationship with the case manager, to support and enable clients to develop skills	
**5. Assertive** **– Intensive case management** **– Recovery** **– Intensive comprehensive care [5,29,53]**	Assertive case management focuses on recovery rather than cure of the health condition (e.g., mental health). It involves; a team providing all necessary treatment and care (at home or work) in their natural environment rather than involving other services; aims to reduce hospitalizations: and purposively outreaches to clients to support their opportunities for choice and living a meaningful and satisfying life as a member of a community. Intensive case management addresses the social and health needs of people, is intensive and long term with an individual case manager.	*Assertive*: Clients are shared by a team to provide services including outreach, direct services such as counselling, skill development, family consultation and support, crisis intervention. Time of involvement is unlimited *Intensive*: small case load which are not shared across the team. *Intensive comprehensive care*: combination of assertive and intensive

*refer to Appendix 2 for details of the articles in scoping study.

The key components of case management described in the papers were extracted. Terms used for these components include activities, functions, tasks, responsibilities, duties, steps and interventions, standards. Across the 79 papers, we mapped 69 of the various terms used in the literature to 17 component headings, which were broadly defined. Only examples of the terms extracted from the literature and mapped to the component are provided in Table 3.

**Table 3:** Examples of the terms in the literature mapped to component heading.

Component Heading	Broad description	Mapped terms

1. Case finding	To identify patients not in contact with services	– Assertive outreach – Detection of patients – Patient identification/outreach – Access – Outreach
2. Establishing rapport	Focusing on the connection developed between the case manager and client Establishing alliance and collaboration with the patient	– Establish and provide a one-to-one relationship – Initial phase – Engagement – Building on the relationship (including with other providers) – Establishing accountability – Establish responsibilities – Negotiate responsibility – Establish therapeutic alliance – Establish long term collaborative and human relationship
3. Assessment	Comprehensive understanding of the needs, capabilities and available resources and community services	– Need identification – Intake – Perform social diagnosis – Assess client and family – Interview – Assessment of needs (e.g. social support, levels of care, readiness and willingness for services, living situation, financial resources, access, barriers, home evaluation, need for referral – Community assessment – Gather information – Use comprehensive assessment instruments – Identify strengths and obstacles to attainment of goals – Cognitive and behavioural assessment – Identify present achievements, interests, resources, interests and aspirations – Document and communicate needs – Document aims and objectives – Estimate level of case management support required – Screening for co-morbid conditions – Determine decision making capacity
4. Planning	Development of plan with client input including setting goals, actions steps towards achievement of goals and selection of resources	– Gatekeeper of funds – Discharge planning – Decision making – Resource identification – Setting goals with client – Goal setting – Design and implementation of care packages – “Moving forward” – Design of an individualized care plan – Determine comparative costs of alternate plan options – Review relapse prevention options – Plan for disengagement of case management
5. Navigation	Facilitate safe and effective connections to services across settings	– Anticipate, identify barriers – Help remove barriers to holistic care
6. Provision of care	Supply care directly or be delegation (relevant to qualifications and experience of case manager)	– Crisis intervention – Patient interventions – Supportive and formal therapeutic interventions – Therapy – Skills training – Patient interventions – Group work – Medication management – Symptom monitoring
7. Implementation	Broker and implement the best package and arrange or purchase services on behalf of the client	– Care arranging – Service implementation – Clinical management – Communication – Arrange and activate services – Develop social networks – Locating and coordinating services – Perform a cost-benefit analysis – Identify formal and informal community resources and support programs – Collect and analyse data – Plan for clients transition along the continuum of care
8. Coordination	Navigating the system of providers and resources needed, referral, facilitate multi-disciplinary collaboration, to ensure and advocate with other agencies for the appropriate use of resources and supports to client, including their purchase of the services themselves.	– Continuity – Linking – Linking to needed services – agency liaison – Environmental interventions – Resource management – Liaison – Facilitation – Interagency coordination – Resource acquisition – Facilitate transitions – Educate and facilitate – Referral – Negotiate – Facilitate patient access – Advocate with providers – Consultation with stakeholders
9. Monitoring		– Proactive support – Monitoring service delivery – Monitor outcomes – Follow-up – Tracking clients – Maintain communication with stakeholders – Monitoring evaluation or reassessment – Maintenance/follow up – “Pushing/pulling and letting go” – Manage
10. Evaluation	Determine the clients progress toward established goals and outcomes and the effectiveness of care	Monitor outcomes and quality of care – Reassessment – Evaluate effectiveness including timeliness – Document client response – Evaluate availability of services needed – Determine. Prepare and communicate when case management services no longer required – Collect and analyse outcome data
11. Feedback		General – Case consultation – Reports to treating providers – Maintain privacy and confidentiality – Regular meetings with treatment team to review goals and progress – Listen to stakeholders, collect information objectively
12. Education/information	Information and assistance to (e.g. client, family other service providers, workplace etc) to assist understanding of e.g. Health condition, Support services	– Providing information – Educate about early signs and symptoms – Assistance with applications, appropriate documents,
13. Advocacy	Advocate for the client, best practice and the payer in line with client’s best interests	– Advocacy for social service programs, during hospitalisation etc – Advocate for more community-based services – Community advocacy – Obtaining financial assistance for the client – Intermittent function, affirmative, assertive approach to assisting client in receiving amenities or services that are being withheld unfairly. – Aiming to have gap/need filled – Assist clients to become autonomous and informed decision-makers
14. Supportive counselling	Provide practical and emotional support, encouragement to facilitate knowledge, coping, adjustment and functioning	– Encouragement/support – Provision of problem solving support – Confrontation – Counselling – Individual, family or social support – Provision of emotional support – Conflict resolution – Provide practical and emotional support
15. Administration	Complete administrative tasks	– Agency and other meetings – Complete paperwork – Treatment planning – Recording, report writing – Audits – Gathering statistics
16. Discharge/Disengagement	Determining and planning for the appropriate time to discontinue case management including facilitating client independence and knowledge to self-manage condition and care needs	– Planning case closure – Case closure
17. Community service development	Support local community to take collective action to develop new, adapt or grow services or generate solutions to common local problems	– Identify gaps – Use of statistics – Prepare funding submission – Create options with generic services – Identify and act on service gaps and overlaps at the client, community and population levels.

## Discussion

The results of the scoping and mapping review confirms that there is a huge body of peer reviewed and grey literature on case management, yet there is significant terminological variance. Following literature searches, exclusions by health conditions, case management context (community-based) and papers with inadequate descriptions we extracted data and mapped the components of case management from papers (n = 79) to the point of saturation. There was a broad range of literature included in the study (quantitative, qualitative, theoretical and practice guidance papers) and from 11 countries and international perspectives (n = 14).

The mapping of extracted data was complicated because of the variability in the language to describe case management. There was heterogeneity in the descriptions, terms and phrases to describe the models, which reflects the difficulties in the articulation of the differences and similarities between the models and the interventions provided by case managers. For the purposes of this scoping review, we mapped the models described to five key models of case management based on a theoretical description of each. Whilst there are more than five case management models, many are variations, adaptations and interpretations of a model to the specific context.

We extracted 69 components in the literature to describe what case managers do (the interventions/activities). We identified 17 key components and mapped the 69 descriptions to these. Each key component had multiple different but related terms to describe the intervention. There was also complexities mapping of the components (activities and interventions) performed by the case manager. In the literature, there was semantic confusion between the components (interventions) of case managers with skills, standards, aims and objectives. For example, ‘stable person- invested but not involved’ [54] is a description of a standard or skill of the case manager (the ‘how’) rather than a component of case management (the ‘what’ is done). The component descriptions were also variously defined from different perspectives of the client, case manager, project or team organisation, program, service or organisation. For example, the description of ‘gatekeeper’ (clinical and financial) [9,55,56] listed as a case manager activity, is aimed at the sustainability of the service or system, at most is an (administrative) responsibility of the case manager to the service or organisation rather than an intervention directed at the client. These difficulties confirm the complexity around case management resulting in terminological variance used. The literature in this scoping study spanned a 25 year period (1988 to 2013). While case management to coordinate services has been used since the late 19^th^ century and contemporary case management emerging since the 1960’s [57], this scoping review confirms that over time the description and terminological variance remains.

The terminological variance reflects the ambiguity and confusion about roles and the interventions performed by case managers. Specificity and replicability of case management are essential to evaluation of effectiveness [58]. There are complex interdependent and dependent factors influencing what case management interventions are done, when, with whom and in what context. A clear understanding and consensus on the components and a common language to describe these factors will provide the tool for measuring outcomes, and making comparisons for effectiveness and quality evaluations.

## Limitations

The study was limited to the descriptions and terms used in the literature to refer to the same or similar concept including the model, theory and components. A limitation in the search strategy was not including all possible databases. Databases such as EMBASE were not searched as it is primarily a biomedical and pharmacological database and considered unlikely to host a significant body of community-based case management literature. Search of the database CINAHL may have revealed additional relevant literature. Whilst other databases could have been considered, the volume of literature from the four databases provided more than sufficient material to use for data extraction to the point of saturation. The extensive search for grey literature added to the volume of peer reviewed literature. However, the point of saturation was reached after the data extraction from 79 articles retrieved through the four databases and multiple grey literature websites.

The search restricted to only four health conditions is a study limitation. The trial of searches without health conditions limits produced in excess of 10,000 hits on Medline alone. For pragmatic reasons, the search strategy was subsequently limited to include four health conditions. Those selected by the authors were known to have community-based case managers involved in health, social care and education sectors.

The study did not undertake quality analysis of the research papers. It is recognised that this meant that equal weight was given to all papers and grey literature, which we consider was justified given the purpose of the scoping study to examine descriptors of case management components and context not efficacy of case management. There can also be concerns about potential bias in scoping reviews related to the reviewers own interests, lack of training and limited view due to discipline or language [59]. Others suggest that there is a ‘trade off’ of potential source of bias in perception and interpretation of a subject and conversely that subject matter experts are necessary [59,60,61,62]. In this instance, considering the complexity in case management, the three researchers background and expert knowledge was considered an advantage to the scoping and conceptual mapping.

The scoping review used five of the six steps in the framework articulated by Arskey et al [17] and extended by Levac [16]. The 6^th^ step it the Arskey/Levac methodology is consultation with a broader group of experts and stakeholders to discuss the findings. This step was not performed as part of the scoping review but did occur in a subsequent step of the larger study to develop a taxonomy on case management. In the larger study, a nominal group of case management experts extensively discussed the results of the scoping review to develop the Beta 2 version of the case management taxonomy [23].

## Conclusion

Case management with all its different names, variations and contexts continues to support the coordination, integration and management of health and social care in many different contexts for different health conditions. The results of this scoping and mapping study confirms the significant terminological variance which produces role confusion, ambiguity and hinders comparability across different health. There is an urgent need for an internationally agreed taxonomy for the coordination, navigation and management of care. The result of this scoping and mapping review was the first of four steps to develop the case management taxonomy finalised in 2015. [23].

## Future research

The results from this scoping and mapping study is part of a larger study to develop a knowledge map and common language, the case management taxonomy which has an intervention tree, service tree and glossary [23].

## Supplementary Files

The supplementary files for this article can be found as follows:

- **Supplementary File 1: Appendix 1. **ijic-16-4-2477-s1.pdf
- **Supplementary File 2: Appendix 2. **ijic-16-4-2477-s2.pdf
- **Supplementary File 3: Appendix 3. **ijic-16-4-2477-s3.pdf
