# Supplementary material for: What Is Case Management? A Scoping and Mapping Review
Source: Int J Integr Care. 2016 Oct 19;16(4):2. doi: 10.5334/ijic.2477 (PMC5388031; doi:10.5334/ijic.2477)
Supplement: Supplementary file 1 [file ijic-16-4-2477-s1.pdf]

Appendix 1 **Search strategy key words**

[‘support broker’ or ‘community care manag*’ or ‘case manag*’ or ‘care monitor*’ or ‘case monitor*’or ‘support coordinat*’ or ‘case coordinat*’ or ‘care coordinat*’ or ‘case management’] AND [definit* or model or Theor* or descript* or describe* or approach* or program evaluation or program develop* or taxonom*] AND [brain injury or head injury or brain trauma or head trauma or brain damage or ABI,mp or TBI.mp] OR [mental health or opioid related disroders or anxiety disorders or depressive disorder or schizophrenia or psychotic disorders or stress, psychological] OR [diabetes mellitus, Type 2 or Type 1, or diabet*] OR spinal cord injuries or spinal cord injury or SCI.mp or quadriplegia or tetraplegia].
