# Supplementary material for: What Is Case Management? A Scoping and Mapping Review
Source: Int J Integr Care. 2016 Oct 19;16(4):2. doi: 10.5334/ijic.2477 (PMC5388031; doi:10.5334/ijic.2477)
Supplement: Supplementary file 2 [file ijic-16-4-2477-s2.pdf]

Appendix 2 Studies included in the scoping review [1-79]

1. Abeyta, N., E.S. Freeman, D. Primack, F.M. Hammond, C. Dragon, A. Harmon, and J. Gassaway, *SCIRehab Project series: the social work/case management taxonomy.* Journal of Spinal Cord Medicine, 2009. **32**(3): p. 336-42.
2. Accident Compensation Corporation. *Glossary of terms - Case manager*. 2013 [cited 2013; Available from: http://www.acc.co.nz/.
3. Ageing Disability and Home Care (NSW). *Case management*. 2013 10 February 2014]; Available from: http://www.adhc.nsw.gov.au/individuals/support/everyday_living_support/what_support_may_benefit_you.
4. Agency for Healthcare Research and Quality, *Care Coordination Measures Atlas*, AHRQ, Editor. 2010: Rockville, USA.
5. Anthony, W.A., M. Cohen, M. Farkas, and B.F. Cohen, *The chronically mentally ill case management--more than a response to a dysfunctional system.* Community Mental Health Journal, 1988. **24**(3): p. 219-28.
6. Applebaum, R. and P. Mayberry, *Long-term care case management: a look at alternative models.* Gerontologist, 1996. **36**(5): p. 701-5.
7. Bedell, J.R., N.L. Cohen, and A. Sullivan, *Case management: the current best practices and the next generation of innovation.* Community Mental Health Journal, 2000. **36**(2): p. 179-94.
8. Bjorkman, T., L. Hansson, and M. Sandlund, *Outcome of case management based on the strengths model compared to standard care. A randomised controlled trial.* Social Psychiatry & Psychiatric Epidemiology, 2002. **37**(4): p. 147-52.
9. Blakely, T.J. and G.M. Dziadosz, *Case management and social role theory as partners in service delivery.* Care Management Journals, 2008. **9**(3): p. 106-12.
10. Brombley, K., *Better at home? Benefits of case management for children with complex needs.* Paediatric Nursing, 2008. **20**(9): p. 24-6.
11. Brubakken, K., S. Grant, M.K. Johnson, and C. Kollauf, *Reflective practice: a framework for case manager development.* Professional Case Management, 2011. **16**(4): p. 170-9; quiz 180-1.
12. Bushy, A., *Case management: considerations for coordinating quality services in rural communities.* Journal of Nursing Care Quality, 1997. **12**(1): p. 26-35.
13. Casarin, S.N., T.C. Villa, R.I. Gonzales, M.C. de Freitas, M.H. Caliri, and C.M. Sassaki, *Case management: evolution of the concept in the 80's and 90's.* Revista Latino-Americana de Enfermagem, 2002. **10**(4): p. 472-7.
14. Case Management Society of America. *Definition of Case Management* 2013 20 August 2013]; Available from: http://www.cmsa.org/.
15. Case Management Society of Australia, ed. *National Standards of Practice for Case Management,*. Third ed. 2013, CMSA.
16. Case Management Society of United Kingdom. *Case Management* 2013.
17. Chamberlain, R. and C.A. Rapp, *A decade of case management: a methodological review of outcome research.* Community Mental Health Journal, 1991. **27**(3): p. 171-88.
18. Cooper, B.J. and D.Y.R. D, *National case management standards in Australia--purpose, process and potential impact.* Australian Health Review, 2006. **30**(1): p. 12-6.
19. Creed, F., B.J. Burns, T. Butler, S. Byford, R. Murray, S. Thompson, and P. Tyrer, *Comparison of intensive and standard case management for patients with psychosis: Rationale of the trial* British Journal of Psychiatry, 1999. **174**(1): p. 74-78.

20. Dieterich, M., C.B. Irving, B. Park, and M. Marshall, *Intensive case management for severe mental illness.* Cochrane Database of Systematic Reviews, 2010(10): p. CD007906.
21. Fiander, M. and T. Burns, *A Delphi approach to describing service models of community mental health practice.* Psychiatric Services, 2000. **51**(5): p. 656-8.
22. Fink-Samnick, E. and L.S. Muller, *Case management across the life continuum: ethical obligations versus best practice.* Professional Case Management, 2010. **15**(3): p. 153-6.
23. Forchuk, C., S. Beaton, L. Crawford, L. Ide, N. Voorberg, and J. Bethune, *Incorporating Peplau's theory and case management.* Journal of Psychosocial Nursing & Mental Health Services, 1989. **27**(2): p. 35-8.
24. Genrich, S.J. and J.S. Neatherlin, *Case manager role. A content analysis of published literature.* Care Management Journals, 2001. **3**(1): p. 14-9.
25. Gensichen, J., M. von Korff, M. Peitz, C. Muth, M. Beyer, C. Guthlin, M. Torge, J.J. Petersen, T. Rosemann, J. Konig, and F.M. Gerlach, *Case management for depresssion by health care assistants in small primary care practices.* Annals of Internal Medicine, 2009. **151**: p. 369-378.
26. Goodwin, D.R., *Nursing case management activities. How they differ between employment settings.* Journal of Nursing Administration, 1994. **24**(2): p. 29-34.
27. Graham, K. and C. Birchmore-Timney, *The problem of replicability in program evaluation. The component solution using the example of case management.* Evaluation & Program Planning, 1989. **12**(2): p. 179-87.
28. Hoeman, S.P. and D.M. Winters, *Theory-based case management: high cervical spinal cord injury.* Home Healthcare Nurse, 1990. **8**(1): p. 25-33.
29. Holloway, F. and J. Carson, *Case management: an update.* International Journal of Social Psychiatry, 2001. **47**(3): p. 21-31.
30. Hosack, K., *Suggestions for case managers who work with patients with severe traumatic brain injury.* Nursing Case Management, 1999. **4**(1): p. 14-8.
31. Hromco, J.G., J.S. Lyons, and R.E. Nikkel, *Styles of case management: the philosophy and practice of case managers.* Community Mental Health Journal, 1997. **33**(5): p. 415-28.
32. Huber, D.L., *The diversity of case management models.* Lippincott's Case Management, 2002. **7**(6): p. 212-20.
33. Jacobson Vann, J., *Measuring community-based case management performance: strategies for evaluation.* Lippincott's Case Management, 2006. **11**(3): p. 147-57; quiz 158-9.
34. Keigher, S.M., *Communication in the evolving world of case management.* Health & Social Work, 2000. **25**(4): p. 227-31.
35. Kennedy, N., J. Barnes, A. Rose, and C. Veitch, *Clinicians' Expectations and Early Experiences of a New Comprehensive Rehabilitation Case Management Model in a Specialist Brain Injury Rehabilitation Unit.* Brain Impairment, 2012. **13**(01): p. 62-71.
36. Kersbergen, A.L., *Case management: a rich history of coordinating care to control costs.* Nursing Outlook, 1996. **44**(4): p. 169-72.
37. Lannin, N., K. Henry, M. Turnbull, M. Elder, and J. Campisi, *An Australian Survey of the Clinical Practice Patterns of Case Management for Clients with Brain Injury.* Brain Impairment 2012. **13**(2): p. 228-237.
38. Lee, D.T., A.E. Mackenzie, S. Dudley-Brown, and T.M. Chin, *Case management: a review of the definitions and practices.* Journal of Advanced Nursing, 1998. **27**(5): p. 933-9.
39. Macan, T., J. Cunningham, M.R. Lemming, and R.J. Calsyn, *Case management and outreach: similarities and differences in worker tasks.* Care Management Journals, 2008. **9**(2): p. 51-62.
40. Marty, D., C.A. Rapp, and L. Carlson, *The experts speak: the critical ingredients of strengths model case management.* Psychiatric Rehabilitation Journal, 2001. **24**(3): p. 214-21.

41. Mas-Exposito, L., J.A. Amador-Campos, J. Gomez-Benito, and L. Lalucat-Jo, *Depicting current case management models.* Journal of Social Work, 2013. **14**(2): p. 133-146.
42. Mas-Exposito, L., J.A. Amador-Campos, J. Gomez-Benito, and L. Lalucat-Jo, *Considering variables for the assignment of patients with schizophrenia to a case management programme.* Community Ment Health J, 2013. **49**(6): p. 831-40.
43. McGrew, J.H., B. Pescosolido, and E. Wright, *Case managers' perspectives on critical ingredients of assertive community treatment and on its implementation.* Psychiatric Services, 2003. **54**(3): p. 370-6.
44. Moore, S.T., *A social work practice model of case management: the case management grid.* Social Work, 1990. **35**(5): p. 444-8.
45. Motor Accidents Authority NSW, *Case management in the NSW Motor Accidents Scheme* I. Management, Editor. 2003, MAA: Sydney
46. Mueser, K.T., G.R. Bond, R.E. Drake, and S.G. Resnick, *Models of community care for severe mental illness: a review of research on case management.* Schizophrenia Bulletin, 1998. **24**(1): p. 37-74.
47. Murray-Leslie, C.F. and C. Kenny, *Case management after severe head injury.* BMJ, 1994. **309**(6957): p. 807.
48. National Case Management Network of Canada. *Standards of Practice for Case Management* 2013 [cited 10 January 2014; Available from: http://www.ncmn.ca/standards.
49. Onus, M., K. McCarthy, I. Nagy, K. Gardner, L. Chenh, M. Doyle, and N. Patel, *Case Management: Is it an intervention or a process?*, in *9th NSW Brain Injury Rehabilitation Forum* 2010: Westmead
50. Oshima, I., N. Cho, and K. Takahashi, *Effective components of a nationwide case management program in Japan for individuals with severe mental illness.* Community Mental Health Journal, 2004. **40**(6): p. 525-37.
51. Parker, M., J. Quinn, M. Viehl, A. McKinley, C.L. Polich, D.F. Detzner, S. Hartwell, and K. Korn, *Case management in rural areas. Definition, clients, financing, staffing, and service delivery issues.* Journal of Nursing Administration, 1992. **22**(2): p. 54-9.
52. Patterson, P.K., H. Maynard, R.M. Chesnut, N. Carney, N.C. Mann, and M. Helfand, *Evidence of case management effect on traumatic-brain-injured adults in rehabilitation.* Care Management Journals, 1999. **1**(2): p. 87-97.
53. Prentice, D., L. Ritchie, M. Reynolds, M. Kitson, J. Smith, and T. Schenck, *A case management experience: implementing best practice guidelines in the community.* Care Management Journals, 2011. **12**(4): p. 150-3.
54. Rapp, C.A., *The active ingredients of effective case management: a research synthesis.* Community Mental Health Journal, 1998. **34**(4): p. 363-80.
55. Rosen, A. and M. Teesson, *Does case management work? The evidence and the abuse of evidence-based medicine.* Australian & New Zealand Journal of Psychiatry, 2001. **35**(6): p. 731-46.
56. Rothman, J., *A model of case management: toward empirically based practice.* Social Work, 1991. **36**(6): p. 520-8.
57. Schaefer, J. and C. Davis, *Case management and the chronic care model: a multidisciplinary role.* Lippincott's Case Management, 2004. **9**(2): p. 96-103.
58. Scheinberg, A.M., W. Gibson, D. Hughes, A. Miles, P. Murphy, and J. Noronha, *Survey of paediatric case management practices in Australia for children and young people with acquired brain injury (ABI).* Child: Care, Health & Development, 2005. **31**(6): p. 679-84.
59. Simpson, G., C. R, A. Gibson, and B. Strettles, *Investigation into the Model of Case Management in the NSW Brain Injury Program* B.I.P.-C.P. Group, Editor. 2010.

60. Solomon, P., *The efficacy of case management services for severely mentally disabled clients.* Community Mental Health Journal, 1992. **28**(3): p. 163-80.
61. Stanton, M.P., M. Swanson, R.A. Sherrod, and D.R. Packa, *Case management evolution: from basic to advanced practice role.* Lippincott's Case Management, 2005. **10**(6): p. 274-84; quiz 285-6.
62. Strassner, L.F., *The ABCs of case management. A review of the basics.* Nursing Case Management, 1996. **1**(1): p. 22-30.
63. Sullivan, W.P. and D.F. Floyd, *There's more than meets the eye: the nuances of case management.* Journal of Social Work in Disability & Rehabilitation, 2012. **11**(3): p. 184-96.
64. Summers, M. and L. Segal, *Evaluatio of the Melbourne City Mission ABI Case Management Service*. 1996, Centre for Health Program Evaluation (Australia),: West Heidelberg,.
65. Tahan, H.A., *Clarifying case management: what is in a label?* Nursing Case Management, 1999. **4**(6): p. 268-78.
66. Tahan, H.A. and V. Campagna, *Case management roles and functions across various settings and professional disciplines.* Professional Case Management, 2010. **15**(5): p. 245-77; quiz 278-9.
67. Tahan, H.A., D.L. Huber, and W.T. Downey, *Case managers' roles and functions: Commission for Case Manager Certification's 2004 Research, Part I.* Lippincott's Case Management, 2006. **11**(1): p. 4-22; quiz 23-4.
68. Thornicroft, G., *Case managers for the mentally ill.* Social Psychiatry & Psychiatric Epidemiology, 1990. **25**(3): p. 141-3.
69. Transport Accident Commission (TAC). *TAC case management model*. 2013 10 February 2014]; Available from: http://www.tac.vic.gov.au/providers/fees-and-policies/policy/supporting/tac-case-management-model.
70. Vuckovic-Kosanovic, J., *Case management and Acquired Brain Injury (ARBIAS service),*, in *Australasian Society for the Study of Brain Impairment (ASSBI)*. 2013: Hobart, Tasmania (Australia).
71. Westmead Hosptial, N., *Case Management - Information for patients*, W.H. Brain Injury Rehabilitation Service, Editor. 2001: Sydney.
72. Williams, M., K. Angstman, I. Johnson, and D. Katzelnick, *Implementation of a care management model for depression at two primary care clinics.* J Ambul Care Manage, 2011. **34**(2): p. 163-73.
73. Wood, J., ed. *External case management of Brain Injury: An overview*. Third edition ed. Rehabilitation, Treatment and Case Management ed. M.J. Ashley. Vol. Chapter 28. 2010, CRC Press, : Florida, USA. 913-932.
74. Wulff, J.J., *Clinical Case Management.* Clinical Case Management, 1991. **23**(2): p. 124-129.
75. Yarmo, D., *Research directions for case management.* Journal of Case Management, 1998. **7**(2): p. 84-91.
76. Yarmo Roberts, D., *Reconceptualizing case management in theory and practice: a frontline perspective.* Health Services Management Research, 2002. **15**(3): p. 147-64.
77. Yarmo-Roberts, D. and J. Stoelwinder, *Untangling the web: the need to clarify care co-ordinating models for people with chronic and complex conditions.* Australian & New Zealand Journal of Public Health, 2006. **30**(5): p. 413-5.
78. Zink, M.R., *Episodic case management in home care.* Home Healthcare Nurse, 2005. **23**(10): p. 655-62.
79. Zwarenstein, M., S. Reeves, S.E. Strauss, P. Pinfold, and J. Goldman, *Case management: effects on professional practice and health care outcomes.* Cochrane Database of Systematic Reviews, 2000(4): p. Art. No.: CD002797. DOI: 10.1002/14651858.CD002797.
