# Supplementary material for: What Is Case Management? A Scoping and Mapping Review
Source: Int J Integr Care. 2016 Oct 19;16(4):2. doi: 10.5334/ijic.2477 (PMC5388031; doi:10.5334/ijic.2477)
Supplement: Supplementary file 3 [file ijic-16-4-2477-s3.pdf]

Appendix 3 Definitions of Case Management

The following specific definitions were found in the published and grey literature.

Case management is:

1. An approach to service delivery that attempts to ensure that clients with complex, multiple problems and disabilities, receive all the services they need in a timely and appropriate fashion Rubin 1987 cited in [1].
2. A way of coordinating, integrating and allocating individualized care within limited resources through by means of continuous contact with one or more key professionals [2].
3. A comprehensive assessment and care planning process involving both the formal and informal system [3].
4. An approach which facilitates assessment and service provision to meet the client's treatment and support needs, and ensures continuity of care throughout the coordination of service delivery across time and setting (Commonwealth Department of Human Services and Health 1995 cited in [1].
5. A collaborative process that assesses, plans, implements, coordinates, and evaluates the options and services required to meet the client's health and human service needs. It is characterized by advocacy, communication, and resource management and promotes quality and cost-effective interventions and outcomes [4].
6. A client focused, collaborative, and educational practice which aims to assist clients to achieve a distinct set of goals within a specific and defined episode of their rehabilitation journey in conjunction with the treating team. This process is managed within a proactive, preventative and responsive problem solving approach [5].
7. A client centred approach involving coordination of multi-disciplinary services within a community that promotes more effective, positive and sustained outcomes for people with an ABI. Active participation and empowerment of the client or the client's designated representative (e.g. guardian) in all aspects of identifying and meeting client/ family needs. Collaborative process that holistically enhances the cognitive, physical, psychosocial, and vocational needs of individuals with acquired brain injury [6].
8. Is the deliberate organization of patient care activities between two or more participants (including the patient) involved in a patients care to facilitate the appropriate delivery of health care services. Organising care involves the marshalling of personnel and other resources needed to carry out all required patient care activities and is often managed by the exchange of information among participants responsible for different aspects of care [7].
9. Is service delivery that fosters client movement through a series of phased involvements within a community's health care systems - facilitate development of community/home base services, enhance efficiency in the delivery of care, ensure quality services, coordinate delivery of needed/appropriate services, target at-risk individuals, prevent admission to institutions and/or acute care facilities, contain costs by controlling access, improve quality of life for client , improve access of services for client, promote continuous participation by client and family, enhance clients health and level of functioning, achieve efficiency [8].
10. Is a collaborative effort which diagnosis, plans, implements, coordinates, supervises and evaluates different options of care according to the health needs of a person through the available resources and communication to promote quality and cost effective results [9].
11. The integration of services on the patient level … someone in the system is taking charge and seeing to it that all the little bits and pieces of the fragmented services system begin to come together in some coherent way. It embodies the concepts of continuity and comprehensiveness in a personalized manner [10].

12. A dynamic and systematic collaborative approach to providing and coordinating healthcare services to a defined population. It is a participative process to identify and facilitate options and services for meeting individual's health needs, while decreasing fragmentation and duplication of care and enhancing quality, cost-effective clinical outcomes. The primary goal of CM is to optimize client functioning by providing quality services in the most efficient and effective manner to individuals with multiple complex needs [11].
13. A collaborative client-driven strategy for the provision of quality health and support services through the effective and efficient use of available resources in order to support the client's achievement of goals [as] related to healthy life and living in the context of the person and their ability. (Canadian Home care association) Case Management is a strategy for maximizing client wellness and autonomy, within their context, through advocacy, communication, education, identification of service resources and service facilitation [12].
14. A comprehensive multi-dimensional assessment of medical, functional, and psychosocial needs; arrangement of community services; coordination across providers; intensive health education and support for lifestyle modifications; and, a methodical tracking of patients' progress between office visits for all recipients regardless of location of where services were provided [13].
15. A collaborative process which assesses, plans, implements, coordinates, monitors and evaluates the options and services required to meet an individual's health, care, educational and employment needs, using communication and available resources to promote quality cost effective outcomes [14].
16. Defined by the Transport Accident Commission as an episodic collaborative process to facilitate the client's achievement of a specified goal, or series of goals over an agreed period of time [15].
17. Often described in the literature as a strategy, a process, and a role. Health care and social service agencies view Case Management as a potential means for improving client care and support. Those who provide Case Management use a collaborative, client-driven process for the provision of quality health and support services promoting the effective and efficient use of resources. Case Management Providers support the clients' achievement of safe, realistic, and reasonable goals within a complex health, social and fiscal environment [16].
18. A collaborative process of assessment, planning, facilitation and advocacy for options and services to meet an individual's health needs through communication and available resources to promote quality cost-effective outcomes [17].
19. Has clinical, strategic and communication roles, is to assist or facilitate resolution of the clients presenting issues, use clinical skills, community resources, client/family capacity to address the issues, it is goal focussed intervention and can be brief, or an episode of planned activity (2-3 years) [18].
20. Aims to ensure that the injured person obtains the services or entitlements that he or she needs. Wherever possible, the aim of providing these services and entitlements to the client is to develop self-sufficiency [19].
21. Is a model and set of technologies for the strategic management of cost and quality outcomes by the clinicians who give the care throughout an entire episode of illness (Zander 1988 cited in [1].
22. The case manager's main role in ABI is to encourage family involvement and participation in rehabilitation, ensure appropriate education about ABI, maximize benefit (insurance) coverage, align team expectations, attend case conferences and validate treatments that are functional and outcome-orientated. Assisting therapists with procuring equipment and anticipating long-term concerns and needs were additionally listed as important factors in the case manager's role (Hosack (1999) cited in [20].
23. A case manager serves as the human link between the client and the system. Its about problem solving. Case management aims to maximise effective use of services and the wellbeing of the

individual. Case management is a way of linking and coordinating services needed so that a client receives the best possible, comprehensive programme of care [21] (Westmead (children's) hospital).

References

1. Yarmo Roberts, D., *Reconceptualizing case management in theory and practice: a frontline perspective.* Health Services Management Research, 2002. **15**(3): p. 147-64.
2. Thornicroft, G., *Case managers for the mentally ill.* Social Psychiatry & Psychiatric Epidemiology, 1990. **25**(3): p. 141-3.
3. Applebaum, R. and P. Mayberry, *Long-term care case management: a look at alternative models.* Gerontologist, 1996. **36**(5): p. 701-5.
4. Case Management Society of America. *Definition of Case Management* 2013 20 August 2013]; Available from: http://www.cmsa.org/.
5. Kennedy, N., J. Barnes, A. Rose, and C. Veitch, *Clinicians' Expectations and Early Experiences of a New Comprehensive Rehabilitation Case Management Model in a Specialist Brain Injury Rehabilitation Unit.* Brain Impairment, 2012. **13**(01): p. 62-71.
6. Vuckovic-Kosanovic, J., *Case management and Acquired Brain Injury (ARBIAS service),*, in *Australasian Society for the Study of Brain Impairment (ASSBI)*. 2013: Hobart, Tasmania (Australia).
7. Agency for Healthcare Research and Quality, *Care Coordination Measures Atlas*, AHRQ, Editor. 2010: ROckville, USA.
8. Bushy, A., *Case management: considerations for coordinating quality services in rural communities.* Journal of Nursing Care Quality, 1997. **12**(1): p. 26-35.
9. Casarin, S.N., T.C. Villa, R.I. Gonzales, M.C. de Freitas, M.H. Caliri, and C.M. Sassaki, *Case management: evolution of the concept in the 80's and 90's.* Revista Latino-Americana de Enfermagem, 2002. **10**(4): p. 472-7.
10. Forchuk, C., S. Beaton, L. Crawford, L. Ide, N. Voorberg, and J. Bethune, *Incorporating Peplau's theory and case management.* Journal of Psychosocial Nursing & Mental Health Services, 1989. **27**(2): p. 35-8.
11. Huber, D.L., *The diversity of case management models.* Lippincott's Case Management, 2002. **7**(6): p. 212-20.
12. Prentice, D., L. Ritchie, M. Reynolds, M. Kitson, J. Smith, and T. Schenck, *A case management experience: implementing best practice guidelines in the community.* Care Management Journals, 2011. **12**(4): p. 150-3.
13. Vann, J.C., *Measuring community-based case management performance: strategies for evaluation.* Lippincott's Case Management, 2006. **11**(3): p. 147-57; quiz 158-9.
14. Case Management Society of United Kingdom. *Case Management* 2013.
15. Transport Accident Commission (TAC). *TAC case management model*. 2013 10 February 2014]; Available from: http://www.tac.vic.gov.au/providers/fees-and-policies/policy/supporting/tac-case-management-model.
16. National Case Management Network of Canada. *Standards of Practice for Case Management* 2013 [cited 10 January 2014; Available from: http://www.ncmn.ca/standards.

17. Case Management Society of Australia, ed. *National Standards of Practice for Case Management,*. Third ed. 2013, CMSA.
18. Onus, M., K. McCarthy, I. Nagy, K. Gardner, L. Chenh, M. Doyle, and N. Patel, *Case Management: Is it an intervention or a process?*, in *9th NSW Brain Injury Rehabilitation Forum* 2010: Westmead
19. Motor Accidents Authority NSW, *Case management in the NSW Motor Accidents Scheme* I. Management, Editor. 2003, MAA: Sydney
20. Scheinberg, A.M., W. Gibson, D. Hughes, A. Miles, P. Murphy, and J. Noronha, *Survey of paediatric case management practices in Australia for children and young people with acquired brain injury (ABI).* Child: Care, Health & Development, 2005. **31**(6): p. 679-84.
21. Westmead Hosptial, N., *Case Management - Information for patients*, W.H. Brain Injury Rehabilitation Service, Editor. 2001: Sydney.
